# Spatial Molecular Architecture of the Microbial Community of a *Peltigera* Lichen

**DOI:** 10.1128/mSystems.00139-16

**Published:** 2016-12-20

**Authors:** Neha Garg, Yi Zeng, Anna Edlund, Alexey V. Melnik, Laura M. Sanchez, Hosein Mohimani, Alexey Gurevich, Vivian Miao, Stefan Schiffler, Yan Wei Lim, Tal Luzzatto-Knaan, Shengxin Cai, Forest Rohwer, Pavel A. Pevzner, Robert H. Cichewicz, Theodore Alexandrov, Pieter C. Dorrestein

**Affiliations:** a Skaggs School of Pharmacy and Pharmaceutical Sciences, University of California, San Diego, California, USA; b Department of Chemistry and Biochemistry, University of California, San Diego, California, USA; c Genomic Medicine, J. Craig Venter Institute, La Jolla, California, USA; d Department of Medicinal Chemistry and Pharmacognosy, College of Pharmacy, University of Illinois at Chicago, Chicago, Illinois, USA; e Department of Microbiology and Immunology, University of British Columbia, Vancouver, Canada; f SCiLS GmbH, Bremen, Germany; g Department of Biology, San Diego State University, San Diego, California, USA; h Natural Products Discovery Group, Department of Chemistry and Biochemistry, Institute for Natural Products Applications and Research Technologies, Stephenson Life Sciences Research Center, University of Oklahoma, Norman, Oklahoma, USA; i European Molecular Biology Laboratory (EMBL), Heidelberg, Germany; j Center for Computational Mass Spectrometry and Department of Computer Science and Engineering, University of California San Diego, La Jolla, California, USA; k Department of Computer Science and Engineering, University of California San Diego, La Jolla, California, USA; l Center for Algorithmic Biotechnology, Institute of Translational Biomedicine, St. Petersburg State University, St. Petersburg, Russia; Pacific Northwest National Laboratory

**Keywords:** lichen, mass spectrometry, microbial assemblages, natural products, metagenomics

## Abstract

Microbes are commonly studied as individual species, but they exist as mixed assemblages in nature. At present, we know very little about the spatial organization of the molecules, including natural products that are produced within these microbial networks. Lichens represent a particularly specialized type of symbiotic microbial assemblage in which the component microorganisms exist together. These composite microbial assemblages are typically comprised of several types of microorganisms representing phylogenetically diverse life forms, including fungi, photosymbionts, bacteria, and other microbes. Here, we employed matrix-assisted laser desorption ionization–time of flight (MALDI-TOF) imaging mass spectrometry to characterize the distributions of small molecules within a *Peltigera* lichen. In order to probe how small molecules are organized and localized within the microbial consortium, analytes were annotated and assigned to their respective producer microorganisms using mass spectrometry-based molecular networking and metagenome sequencing. The spatial analysis of the molecules not only reveals an ordered layering of molecules within the lichen but also supports the compartmentalization of unique functions attributed to various layers. These functions include chemical defense (e.g., antibiotics), light-harvesting functions associated with the cyanobacterial outer layer (e.g., chlorophyll), energy transfer (e.g., sugars) surrounding the sun-exposed cyanobacterial layer, and carbohydrates that may serve a structural or storage function and are observed with higher intensities in the non-sun-exposed areas (e.g., complex carbohydrates).

**IMPORTANCE** Microbial communities have evolved over centuries to live symbiotically. The direct visualization of such communities at the chemical and functional level presents a challenge. Overcoming this challenge may allow one to visualize the spatial distributions of specific molecules involved in symbiosis and to define their functional roles in shaping the community structure. In this study, we examined the diversity of microbial genes and taxa and the presence of biosynthetic gene clusters by metagenomic sequencing and the compartmentalization of organic chemical components within a lichen using mass spectrometry. This approach allowed the identification of chemically distinct sections within this composite organism. Using our multipronged approach, various fungal natural products, not previously reported from lichens, were identified and two different fungal layers were visualized at the chemical level.

## Abstract (precis)

Microbial communities have evolved over centuries to live symbiotically. The direct visualization of such communities at the chemical and functional level presents a challenge. Overcoming this challenge may allow one to visualize the spatial distributions of specific molecules involved in symbiosis and to define their functional roles in shaping the community structure. In this study, we examined the diversity of microbial genes and taxa and the presence of biosynthetic gene clusters by metagenomic sequencing and the compartmentalization of organic chemical components within a lichen using mass spectrometry. This approach allowed the identification of chemically distinct sections within this composite organism. Using our multipronged approach, various fungal natural products, not previously reported from lichens, were identified and two different fungal layers were visualized at the chemical level.

## INTRODUCTION

What forces a microbial assemblage to function as one unit and survive in a harsh environment is a challenging question to address. In order to answer this question, one must consider that assemblages typically include different domains of life. The different organisms can be uniquely spatially organized within a community and thus form an orchestrated chemical interactome map that is also influenced by abiotic factors, such as sun exposure. Many of the small molecules that make up this chemical environment are involved in interactions between the community members influencing overall community homeostasis and survival (1–4). It remains a challenge to create an inventory and to identify the organismal sources of the small molecules mediating these community-wide chemical interactions and to study their spatial distribution.

Lichens represent one such complex organism consisting of all three domains of life living inside and outside the thallus, i.e., the lichen body. This complex community provides myriad opportunities for symbiotic interactions and is recognized as a potential source of pharmaceutical drug discovery (5–8). Initially, lichens were thought to be composed mainly of a photobiont (cyanobacteria or algae) and a mycobiont (fungi) (9). Later, culture-independent methods showed the presence of various nonphototrophic bacterial species (10–13), and recently, a third partner embedded in the lichen cortex or “skin”—basidiomycete yeasts (single-celled fungi) (14)—was reported and challenged the established one-fungus–one-lichen symbiosis present in lichen. The symbiosis between these different domains of life allows lichens to survive in extremely nutrient-poor environments and also provides protection from insects and invasion by other life forms. Differential metabolite production was observed in specific regions (cortex, medulla, and soralia) of *Roccella* sp. lichen by using thin-layer chromatography and mass spectrometry (MS) (15), but the direct analysis of spatial distribution in a continuous piece of lichen has not been performed. Lichen’s naturally complex consortia of distinct life forms are likely behaving differently when community members are maintained in pure monocultures, and the metabolic exchange between these consortia across different layers of the lichen remains unexplored.

Various mass spectrometry (MS)-based methods such as matrix-assisted laser desorption ionization imaging mass spectrometry (MALDI IMS), desorption electrospray ionization (DESI) IMS, and secondary ion MS allow visualization of biological compounds directly from a biological sample (16–19). Optical imaging and fluorescence microscopy-based methods such as catalyzed reporter deposition fluorescence *in situ* hybridization (CARD-FISH) generate images that can be used to evaluate the microbial populations of ecosystems and multicellular tissues (20–22). Sequencing-based methods such as deep sequencing of rRNA genes, internal transcribed spacer (ITS) regions, and metagenomic sequencing (11, 23) allow for the taxonomic identification of microbial taxonomic units in communities. While these methods are powerful, they do not address how individual chemical contributions influence the lichen-associated community. In this study, we utilized MALDI IMS and orthogonal tandem MS (MS/MS)-based molecular networking to visualize the distributions of biological compounds and annotate microbial chemistry in order to define their biological origin, respectively. The mass spectrometry-based analyses were complemented with metagenomic sequencing to characterize the taxonomic composition and to link compounds to biosynthetic gene clusters.

## RESULTS AND DISCUSSION

The *Peltigera* sp. lichens are commonplace in humid environments and are mainly found living on soils in forests and along roadsides (24). Here, we obtained a 1.8-cm by 3-cm piece of *Peltigera hymenina* lichen, and prior to spatial metabolomics analyses, we first determined its taxonomic composition and the genetic potential to produce small molecules and isolated and cultured microbial members. This allowed us to assess taxonomic community signatures and the functional architecture from a gene-to-molecule prediction standpoint. By analyzing the relative abundance of taxonomically annotated genes as outlined in Materials and Methods, the lichen community was found to be composed of 80.9% bacteria (of which 5.2% were *Cyanobacteria*), 0.001% archaea, and 18.8% eukaryota (of which 17.1% were fungi). The abundance of eukaryotic and bacterial taxa is presented in Fig. S1 in the supplemental material. In previous studies using confocal laser scanning microscopy, millions of bacterial cells forming structured assemblies per gram of lichen thallus have been identified (11, 12). We observed that these assemblies are highly diverse, consisting of hundreds of taxa. Only a few archaea were detected from annotated genes, which is consistent with previous findings (13). The major bacterial phyla classified in this study were *Proteobacteria*, *Bacteroidetes*/*Chlorobi* group, *Actinobacteria*, *Cyanobacteria*, and *Fibrobacteres*/*Acidobacteria* group (Table 1).

10.1128/mSystems.00139-16.2 Figure S1  Pie charts representing taxonomic diversity of bacterial (A) and fungal (B) genes associated with the lichen under study. Bacterial taxa are presented at the phylum level, and fungal taxa are presented at the species level. Taxonomic annotation of genes was performed using the METAREP Web tool. Download Figure S1, EPS file, 0.8 MB. Copyright © 2016 Garg et al. 2016 Garg et al. This content is distributed under the terms of the Creative Commons Attribution 4.0 International license.

**TABLE 1:** Eukaryotic species and bacterial phyla associated with the lichen under study^a^

Organism^b^	Absolute gene count	Relative gene count (%)
Eukaryota (species level)		
* Ajellomyces capsulatus* (A)	5,494	11.9
* Talaromyces stipitatus* (A)	4,361	9.9
* Arthroderma gypseum* (A)	1,959	4.2
* Botryotinia fuckeliana* (A)	1,687	3.6
* Cryptococcus neoformans* (B)	1,132	2.4
* Geomyces destructans* (A)	797	1.7
* Sclerotinia sclerotiorum* (A)	746	1.7
* Rhizopus oryzae* (O)	698	1.5
Unresolved fungus	4,474	9.7
Bacteria (phylum level)		
* Proteobacteria*	102,582	51.4
* Bacteroidetes*/*Chlorobi*	35,465	17.7
* Actinobacteria*	20,176	10.1
* Cyanobacteria*	10,405	5.2
* Fibrobacteres*/*Acidobacteria*	10,311	5.2
* Chlamydiae*/*Verrucomicrobia*	6,220	3.1
* Planctomyces*	6,213	3.1
* Firmicutes*	2,813	1.4

a Taxonomic predictions were derived from assembled metagenome annotations and relative gene counts by using the JCVI-supported METAREP analysis pipeline. Taxonomic units contributing >1% to the total community abundance are presented.

b A, Ascomycetes; B, Basidiomycetes; O, other fungus.

Within the *Cyanobacteria* phylum, *Nostoc punctiforme* contributed 40.5% to the total cyanobacterial abundance, showing the typical bacterial taxonomic signature for *P. hymenina* (25). The presence of *N. punctiforme* was verified by mapping metagenomic reads to the *Nostoc*-specific RuBisCO (*rcbLX*) (e.g., NCBI accession numbers KJ413212.1 and DQ185279) and genes encoding nosperin (*nspA* and *nspF*) (NCBI accession number GQ979609.2) using reference genes and genomes available in GenBank. Eukaryotic genes were most similar to species within the Ascomycetes (Table 1). These matches likely represent genes belonging to *P. hymenina*, for which no genome sequence is currently available. The identity of *P. hymenina* was further confirmed by mapping metagenomic reads to the *P. hymenina* internal transcribed spacer (ITS) sequence, which was previously obtained from the studied specimen by PCR and sequencing (NCBI accession number KX790924). The assembled sequence showed high sequence identity (99%) to *Peltigera hymenina*. The metagenomic contig sequences showed a high rank score (0.9 to 1) at the kingdom level, indicating that they could be classified with high certainty as either bacteria, archaea, virus, or Eukaryota (see Table S1 posted at ftp://massive.ucsd.edu/MSV000078584/updates/2016-11-18_zengyi88516_edceffc7/other/). In summary, the mycobiont part of the lichen was most closely related to *P. hymenina* and the remaining organisms corresponded to a total of 324 phyla of which 74 represent viruses, 236 represent bacteria, 8 represent eukaryotes, and 6 represent archaea (see Table S1 available at ftp://massive.ucsd.edu/MSV000078584/updates/2016-11-18_zengyi88516_edceffc7/other/). A surprisingly high number of viral genes were identified, which has not been reported previously in association with a lichen. At the species level, approximately 40% of the virus contigs had low rank scores (<0.5), indicating that they are missing database representatives. Two contigs (43,483 bp and 35,832 bp long, respectively) harbored the lytic phage *Lactococcus* phage bIL312 genome (genome size, ~15 kb) (see the supplemental material and see also Table S1 available at ftp://massive.ucsd.edu/MSV000078584/updates/2016-11-18_zengyi88516_edceffc7/other/). Additional discussion on metagenomics-based taxonomy analysis is provided in Text S1 and Fig. S2 in the supplemental material.

10.1128/mSystems.00139-16.1 Text S1  Supplemental materials and methods. Download Text S1, DOCX file, 0.04 MB. Copyright © 2016 Garg et al. 2016 Garg et al. This content is distributed under the terms of the Creative Commons Attribution 4.0 International license.

10.1128/mSystems.00139-16.3 Figure S2  Hierarchical cluster analysis of taxonomic annotations of genes and their relative frequencies from metagenomes representing a diversity of environments (lichen annotations boxed in red). Metagenome annotations and relative abundance values are available via the J. Craig Venter Institute through the JCVI-supported METAREP (http://www.jcvi.org/hmp-metarep). Colors represent relative percentages of total annotated genes belonging to orders of bacteria (B), fungi (F), and eukaryotes (other than fungi) (E). Light pink to red, >3 to 14%; white, 3%; light blue to blue, 0.1 to <3%. Pearson correlation distance is shown next to cluster topography. Download Figure S2, EPS file, 1.3 MB. Copyright © 2016 Garg et al. 2016 Garg et al. This content is distributed under the terms of the Creative Commons Attribution 4.0 International license.

To gain insights into the genetic potential of *P. hymenina* lichen to synthesize small molecules, all identified genes from the assembled contigs were subjected to annotation by using Kyoto Encyclopedia of Genes and Genomes (KEGG) categories, which are a part of the JCVI in-house METAREP prokaryotic pipeline (http://www.jcvi.org/metarep/) (26). METAREP showed the highest numbers of gene matches to xenobiotic biodegradation and metabolism (72,471 matches), biosynthesis of other secondary metabolites (69,809 matches), metabolism of terpenoids and polyketides (68,677 matches), lipid metabolism (54,404 matches), glycan biosynthesis and metabolism (50,131 matches), amino acid metabolism (41,767 matches), metabolism of cofactors and vitamins (36,167 matches), and carbohydrate metabolism (40,798 matches) (see Fig. S3 in the supplemental material). Overall, 13% (i.e., 75,076 genes) of all identified genes were broadly classified as enzymes involved in production of secondary metabolites such as terpenes, flavones, nonribosomal peptides, and polyketides, the types of molecules identified by our mass spectrometry workflows (see below).

10.1128/mSystems.00139-16.4 Figure S3  Kyoto Encyclopedia of Genes and Genomes (KEGG) pathway-level annotations and relative counts of assembled genes representing the lichen metagenome. The JCVI-supported METAREP (http://www.jcvi.org/hmp-metarep) tool was used for classification. Numbers of total gene counts that could be classified for each functional group are presented inside brackets. Download Figure S3, EPS file, 1.5 MB. Copyright © 2016 Garg et al. 2016 Garg et al. This content is distributed under the terms of the Creative Commons Attribution 4.0 International license.

**FIG 1:**
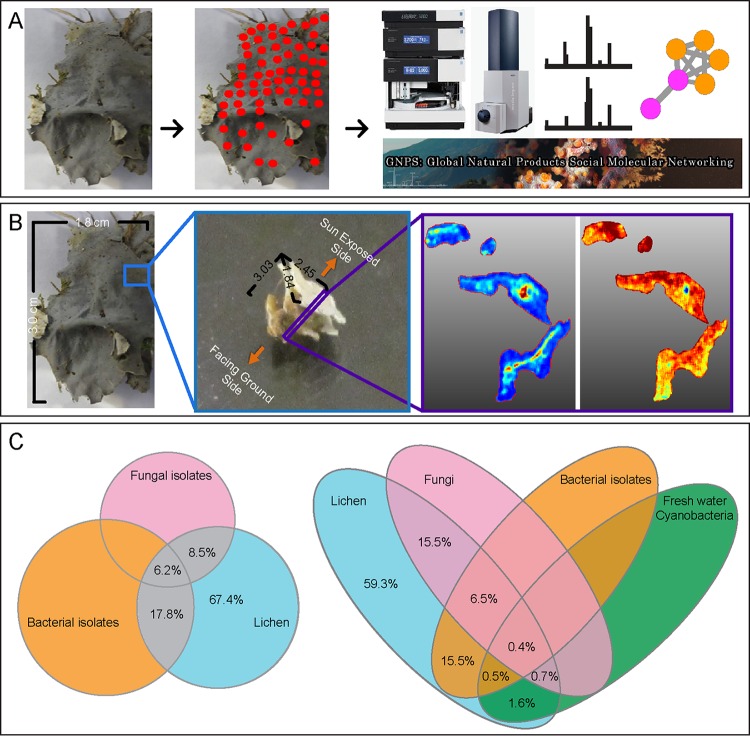
(A) A needle was used to scrape off small amounts of material from 110 locations from the original piece of lichen. UHPLC-MS/MS data were acquired on these materials, and data analysis was performed using the online analysis infrastructure GNPS. (B) A 2.5-mm by 1.8-mm by 3-mm piece of lichen was sectioned from the original lichen piece (1.8 cm by 3 cm). This section was embedded in gelatin, and MALDI IMS data were acquired on three layers, the sun-exposed layer, the middle layer, and the bottom layer, to reveal metabolite distributions in false color. (C) The Venn diagram on the left shows the percentage of molecules detected in lichen that are of either fungal or bacterial origin. This Venn diagram is scaled to demonstrate the number of features detected. The origin was assigned by identifying common molecules in the MS/MS data acquired on the lichen and microbes cultured from this lichen. The Venn diagram on the right shows percentages of common molecules among lichen, cultured microbial isolates, and public data sets on soil fungi as well as freshwater cyanobacteria.

Next, we employed an untargeted MS-based metabolomics approach by using the crowdsourcing-based Global Natural Product Social (GNPS) molecular networking infrastructure, which supports high throughput and is freely available (27). Since GNPS captures MS knowledge from the community, including MS data from microbes, the spectral diversity available is appropriate to aid identification in our untargeted MS data (27). Here, to enable the mass spectral analysis from *Peltigera* sp. lichen, a small amount of material was scraped off using a needle from 110 spots from the 3-cm by 1.8-cm section of lichen (Fig. 1A). The metabolites from these samples were extracted with organic solvents and analyzed by ultrahigh-performance liquid chromatography (UHPLC) coupled to quadrupole time of flight (Q-TOF) MS and subjected to tandem MS. To further define the molecular contributions of the community, bacteria, cyanobacteria, and fungi were cultured from the same lichen specimen prior to genomic and mass spectrometry analyses. The metabolite extractions from the agar-grown cultures of isolated microbes were performed under the identical conditions as the 110 scrapes and subjected to the same UHPLC-MS/MS workflows. The MS/MS spectra acquired from the lichen and the microbial cultures were coanalyzed using the GNPS infrastructure to identify the known small molecules present with the reference MS/MS spectra as well as to match the lichen MS/MS spectra with the microbe MS/MS spectra using molecular networking. The distributions of these molecules were visualized using MALDI IMS (Fig. 1B). These orthogonal MS-based analyses are described below.

The UHPLC-MS-detectable chemical space of the *Peltigera* sp. lichen was explored using molecular networking. The gas phase fragmentation of molecules in tandem MS is dictated by their chemical structure and is recorded in the form of MS/MS spectra that can be thought of as “chemical barcodes.” Molecular networking has been employed to dereplicate (annotate known molecules based on nearly identical chemical barcodes) the detected molecular families and to assign compound production to specific community members (28–33). Furthermore, when a spectral match is common to the data collected directly from the lichen and the cultivable individual members, such as the bacteria, cyanobacteria, and fungi, one can trace chemical contributions by the individual members forming the community. The matched spectra are displayed in the form of nodes where each node represents a single molecule and contains underlying MS/MS spectra originating from either the lichen, the isolated microbes, or both. The nodes that are connected to each other represent molecules that are structurally related and hence have similar fragmentation patterns or chemical barcodes. This structural (fragmentation) similarity is quantified by a cosine score between the MS/MS spectra which is visualized as the thickness of the lines (edges) connecting the nodes. The UHPLC-MS/MS spectra collected from the 110 different scrapes of the *P. hymenina* lichen and the agar-grown cultures of isolated microbes were aligned and represented in the form of molecular networks using the GNPS crowdsourced analysis infrastructure with the goal of gaining insight into the diversity of chemistry associated with this lichen (see Fig. S4 in the supplemental material).

10.1128/mSystems.00139-16.5 Figure S4  The molecular network of MS/MS data collected on 110 regions from a lichen piece and cultured bacterial and fungal isolates. The nodes in orange are molecules detected only in lichen, the nodes in pink are molecules detected both in lichen and in microbial cultures, and the nodes in purple are molecules detected only in cultured microbes. Download Figure S4, TIF file, 2.2 MB. Copyright © 2016 Garg et al. 2016 Garg et al. This content is distributed under the terms of the Creative Commons Attribution 4.0 International license.

The network analyses revealed that 8.5% and 17.8% of the nodes with the origin matched to cultured microbes were of fungal and bacterial origin, respectively (Fig. 1C). In order to increase assignments of the origin of the MS/MS data, the MS/MS data from publicly available MassIVE data sets from freshwater cyanobacteria and soil and skin fungi (MassIVE accession numbers MSV000079029, MSV000079098, and MSV000078666, respectively) were aligned with the existing MS/MS data on the lichen and the cultured microbes. The public data set MSV000079098 was acquired on fungal extracts prepared from a collection of a large number of diverse sources as a part of a citizen science project (http://npdg.ou.edu/citizenscience) (34). The resulting molecular network showed an increase in assignment of fungus-specific molecules from 8.5% to 15.5% (Fig. 1C). Conetworking with public freshwater cyanobacterial data allowed us to assign 1.6% of the lichen molecules to cyanobacteria. The MS/MS spectral matches were more abundant for bacteria, in agreement with the sequence alignments detected with the metagenomic analysis. Although a large portion of chemistry still remains unidentified, these observations highlight that isolation of individual community members and use of existing and growing chemical knowledge in the public databases can provide deeper insights into community compositions. To the best of our knowledge, this is one of the first instances where public metabolomics data (not reference spectra) have been reused to dereplicate newly acquired data in a completely different analysis. Furthermore, using the GNPS reference libraries, many molecules could be annotated. The putative annotations are described in detail in the supplemental material under annotation of molecules by MS and follow the “level 2” annotation standard as proposed by the Metabolomics Society standard initiative (35). The network statistics from the GNPS output revealed that 0.7% of the nodes in the molecular network were dereplicated, which is in agreement with the 1.8% average annotations possible with untargeted metabolomics analyses and includes many molecules that have not been reported to be made by lichens, such as PF1140, asperphenamate, sesquiterpene lactones, and cyanobacterial glycolipids. Annotations of these molecules enabled visualization of distinct fungal and cyanobacterial layers using MS-based imaging (described below).

**FIG 2:**
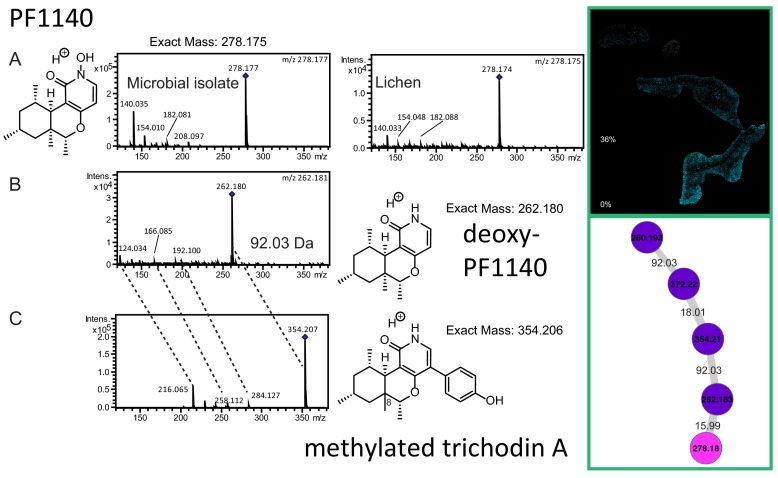
The molecular family belonging to fungal pyridone alkaloid PF1140 is shown. (A) The representative molecule PF1140 was identified in both lichen and fungal isolate (pink node) as well as in the MALDI IMS data. (B) The cultured microbes also produced a previously known analogue, deoxy-PF1140 (purple node). (C) The isolated microbe also showed production of an unknown molecule at *m/z* 354.207 (purple node). The shift in the parent mass for this unknown molecule by 92.03 Da from the mass of deoxy-PF1140 suggested addition of a phenol group to deoxy-PF1140. The MS/MS fragments also showed a shift of 92.03 Da in mass (shown in dashed lines). A structure search in SciFinder suggested the molecule to be a new analogue of trichodin A with one additional methyl group. The putative structures and the tandem MS spectra of other molecules in this cluster are shown in Fig. S5 in the supplemental material.

**FIG 3:**
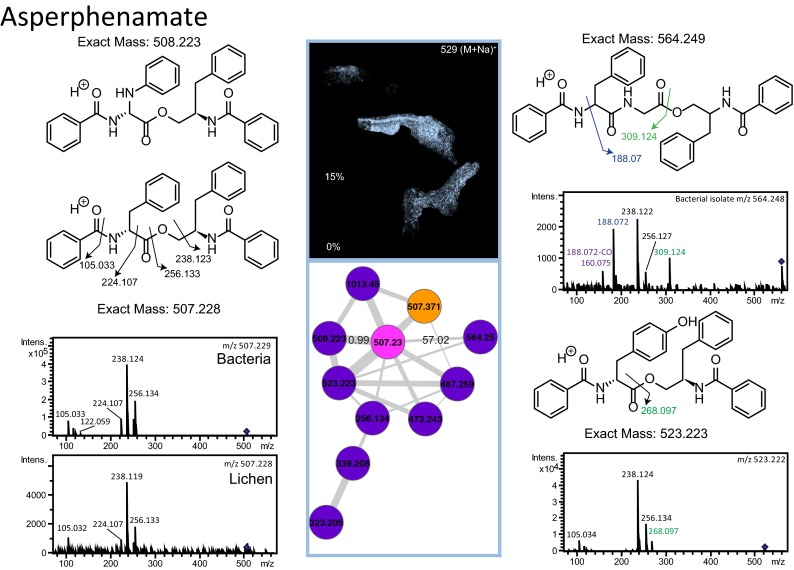
The molecular network of the fungal molecule asperphenamate (*m/z* 507.229) and its distribution are shown. The pink node represents a common molecule produced by both the lichen and the isolated microbe. The purple nodes are unique to the cultured microbe, and the orange node is unique to lichen. The underlying MS/MS spectra for asperphenamate in the orange node were recorded at lower intensity and also have a contaminating MS/MS spectrum from a molecule with similar mass. Hence, this node is not merged with the pink node corresponding to asperphenamate. The MS/MS spectra of asperphenamate (*m/z* 507.229) and the analogues (*m/z* 532.222 and 564.248) are annotated in the MS/MS spectra shown, and annotations are described in Text S1 in the supplemental material.

To provide spatial insight into the molecules that are present in the lichen layers, a 2.5-mm by 1.8-mm by 3-mm block of *P. hymenina* lichen that included 3 layers of lichen, one of which was sun exposed, was embedded in gelatin, frozen, and sectioned using a cryostat (Fig. 1B). MALDI IMS data were collected on all three layers (sun-facing top layer, middle layer, and bottom layer) as described in Materials and Methods. The molecular distribution of individual molecules in the *P. hymenina* lichen sun-exposed top layer, middle layer, and bottom layer was constructed using false colors and analyzed to decipher spatial chemical exchange between community members. A sessile community of organisms, such as a lichen, can take years to develop and needs to defend itself from invasive species and predators. This is reflected by the presence of defense molecules that were found throughout the lichen, albeit with different distributions. While these molecules are believed to play a role in defense against pathogens, we hypothesize that since these distributions are observed not only at the surface but also elsewhere, they play a role in how the community is shaped. The distributions of the fungal molecules asperphenamate and PF1140 are observed in different locations inside the lichen (Fig. 2 and 3; see also Fig. 8A). The fungal pyridone alkaloids, such as PF1140, have a wide range of known biological activities, including antifungal and antibacterial activities (36–38). Asperphenamate is a peptidic natural product with anticancer activities and had been previously isolated from *Penicillium* sp. and *Aspergillus* sp. (39–41), but its production has not yet been reported from lichens. However, the presence of *Aspergillus* and *Penicillium* sp. was not indicated by metagenomic sequencing results (Table 1). Furthermore, asperphenamate was detected in 33 different fungal extracts analyzed as part of the citizen science project (MassIVE data set MSV000079098 described above), and thus, these molecules likely represent a common fungal defense mechanism available to various fungi. The highest abundance of PF1140 was present in the bottommost layer, which has the least exposure to sun (Fig. 2), whereas the asperphenamate (Fig. 3) was mostly concentrated in the middle layer and a lower abundance was present in the top layer surrounding the cyanobacterial layer (see below for cyanobacterial layer). In addition, PF1140 was distributed more on the periphery of the lichen specimen, whereas asperphenamate is present inside the lichen (see Fig. 8A). It is likely that these molecules are therefore produced by two different fungal species in this lichen. This observation is interesting in light of recent work showing additional presumptive symbionts, basidiomycete yeasts, associated with a variety of lichens (14). Indeed, the MS/MS spectra for these molecules belong to two distinct fungal isolates cultured from the lichen in this study. Thus, differential distribution of PF1140 and asperphenamate and the observation that they are produced by two different isolates support the hypothesis that distinct fungal species are present in the region where asperphenamate and PF1140 are present. These molecules with antimicrobial properties cover the entire lichen imaged in this study, giving rise to a protective advantage against invading pathogens provided to the lichen community by fungi. In many ways, this resembles multicellular defense strategies seen in tissues from higher eukaryotes as well.

10.1128/mSystems.00139-16.6 Figure S5  Putative structures and MS/MS spectra of two fungal molecules detected in the pyridone alkaloid PF1140 family. The molecule on the left has the identical parent *m/z* as deoxyakanthomycin, and the molecule on the right is 92.03 Da in mass higher than deoxyakanthomycin. This molecule likely represents a new analogue of deoxyakanthomycin with a phenol moiety on the pyridine ring. This phenol group is also present on other known pyridine alkaloids such as trichodin A. Download Figure S5, EPS file, 0.4 MB. Copyright © 2016 Garg et al. 2016 Garg et al. This content is distributed under the terms of the Creative Commons Attribution 4.0 International license.

**FIG 4:**
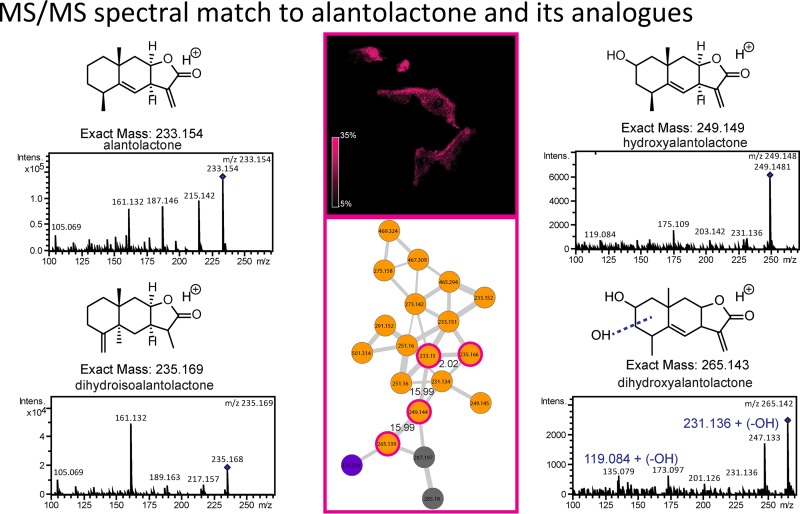
The molecular network of a family of sesquiterpene lactones and the distribution of a representative molecule with an MS/MS spectrum match to alantolactone are shown. All the labeled MS/MS peaks for alantolactone matched the MS/MS spectra available on the METLIN metabolite database. The known analogues at *m/z* 249.149 and 235.169 are annotated as hydroxyalantolactone and dihydroisoalantolactone based on the MS/MS data available on METLIN. The molecule at *m/z* 265.143 is annotated as dihydroxyalantolactone due to an increase in the parent mass of 15.99 Da from the mass of hydroxyalantolactone. The corresponding fragments with a 15.99-Da shift are labeled in the MS/MS spectra in blue. Orange represents spectra found in lichen samples only, pink represents spectra found in both lichen and cultured isolates, purple represents spectra detected only in cultured isolates, and grey nodes represent other combinations.

Another annotated molecular family includes sesquiterpene lactones. The MS/MS spectral similarity to alantolactone (Fig. 4) and its analogues hydroxyalantolactone, dihydroisoalantolactone, and dihydroxyalantolactone suggested the presence of this molecular family in lichens. Alantolactone has been isolated from medicinal plants and displays a myriad of biological activities such as antimicrobial, anticancer, antiparasitic, and anti-inflammatory properties (42–44). This molecule displayed a distribution similar to that of the fungal molecule asperphenamate in lichen and is present mainly inside the lichen (see Fig. 8A). Lichens were previously not known to contain this molecule, and the distribution of the sesquiterpene alantolactone-like molecule suggests that it may be produced by a fungus and may constitute the protective layer either from the external environment or from fungi present in the lichen community. The production of this molecule was not observed in cultured isolates.

**FIG 5:**
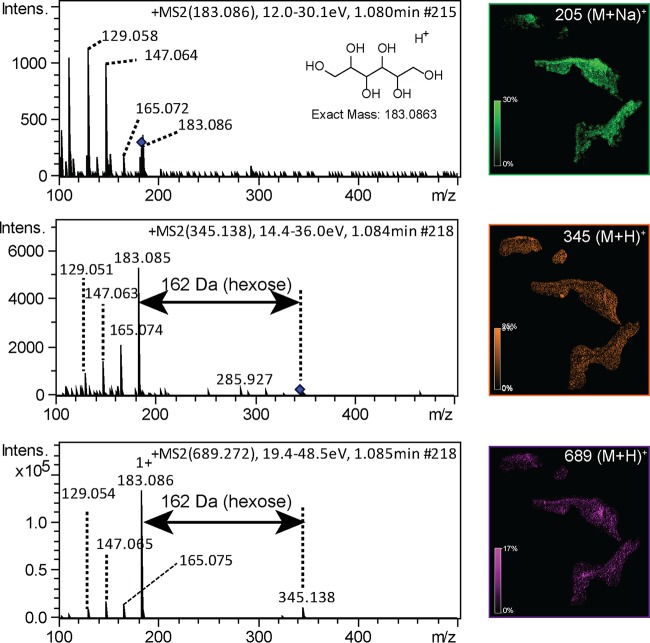
The MS/MS spectra and MALDI IMS distributions of mannitol and the corresponding polysaccharide containing mannitol are shown. The polysaccharide at *m/z* 345.138 contains an additional hexose residue, and the polysaccharide at *m/z* 689.272 contains two additional hexose sugar residues. The molecular network corresponding to the polysaccharide family is shown in Fig. S6B in the supplemental material.

Earlier studies show that simple sugars or polyols produced by the cyanobacteria are converted by fungi into biomass or utilized as energy sources. The movement of sugars, such as glucose or ribitol, has been previously observed from the photobiont to fungi, which convert it rapidly to mannitol, allowing fungi to store additional water required by both the fungi and the cyanobacteria under dry conditions (45, 46). Fungi in lichens also produce various polysaccharides that may play a role as nutrient sources in metabolically inactive states or as protective agents and have been associated with the medicinal properties of lichen (47–49). Various polysaccharides were observed by molecular networking (see Text S1 and Fig. S6 in the supplemental material). The distribution of the mannitol-containing sugar family is shown in Fig. 5. Mannitol is mainly localized to the middle and bottom layers, similarly to the distribution of the fungal molecules PF1140 and asperphenamate. The mannitol-containing polysaccharides at *m/z* 345.138 and 689.272 have identical distributions, an indication that they are produced by the same organism(s) and are likely part of the same biosynthetic pathway. The polyacetylated polysaccharides are represented in most of the lichens (see Fig. S6C and S7 in the supplemental material), suggesting a potential for these polysaccharides to be of structural nature or to serve as energy storage in the same way that glycogen is stored as a polysaccharide. Distribution of the polyacetylated polysaccharide family overlapped the distribution of UDP-*N*-acetylglucosamine, an acetylated sugar (see Fig. S7). Thus, the colocalization of these polysaccharides and UDP-*N*-acetylglucosamine appears to reflect the metabolically active areas of the lichen.

10.1128/mSystems.00139-16.7 Figure S6  (A) The delta *m/z* histogram obtained from GNPS analysis of the molecular network is shown (http://gnps.ucsd.edu/ProteoSAFe/result.jsp?task=675991870933480293c10f0bfcf69e20&view=pairs_histogram). This analysis is obtained under the network summarizing graphs tab on the results page. Common delta *m/z* shifts of sugar residues between parent masses are labeled. The *y* axis represents the number of pairs in the network that have these shifts, and the *x* axis represents the delta *m/z* shift itself. (B) The molecular network of the polysaccharide family is shown and is accessible online at http://gnps.ucsd.edu/ProteoSAFe/result.jsp?view=network_displayer&componentindex=224&task=675991870933480293c10f0bfcf69e20#%7B%7D, where delta *m/z* between nodes can be highlighted for visualization purposes. The nodes in orange are unique to lichen, the nodes in purple are unique to microbial isolates, and the nodes in gray are polysaccharides that are also present in the culture medium. (C) The molecular network of the polyacetylated polysaccharide family is shown. The nodes differ by 42.01 Da and 84.02 Da (2 × 42.01) in mass, indicative of polyacetylated residues. Download Figure S6, EPS file, 2.2 MB. Copyright © 2016 Garg et al. 2016 Garg et al. This content is distributed under the terms of the Creative Commons Attribution 4.0 International license.

10.1128/mSystems.00139-16.8 Figure S7  The distributions and MS/MS spectra of UDP-*N*-acetylglucosamine and the polyacetylated polysaccharide are shown. The polysaccharide contains a hexose sugar residue (162 Da) and glucuronic acid (176 Da) as well as polyacetylations (*n* × 42.01) on sugar residues. Download Figure S7, TIF file, 1.2 MB. Copyright © 2016 Garg et al. 2016 Garg et al. This content is distributed under the terms of the Creative Commons Attribution 4.0 International license.

**FIG 6:**
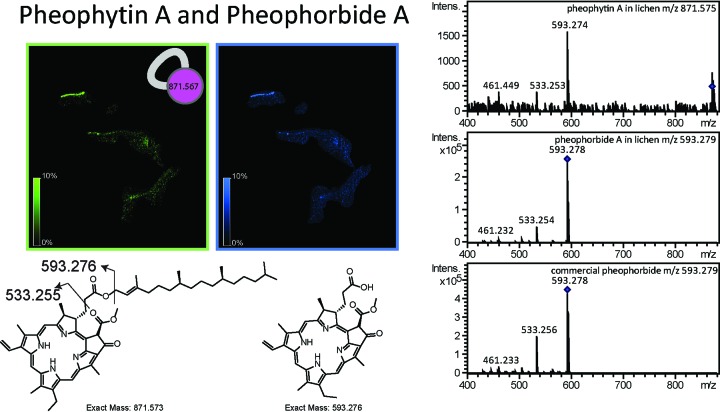
The chlorophyll *a* pigments pheophytin A and pheophorbide A were identified in both UHPLC-MS/MS data and MALDI-IMS data. The two major fragments at *m/z* 593.276 and *m/z* 533.255 are annotated in the structure of pheophytin A.

**FIG 7:**
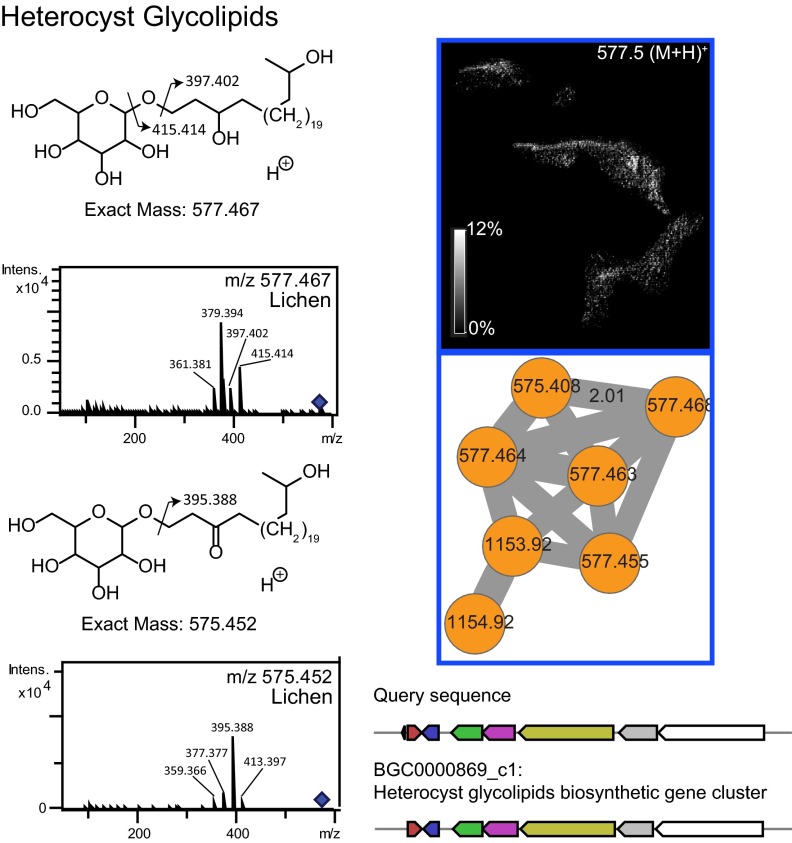
The mass spectrum of heterocyst glycolipid and the corresponding structures are shown on the right. The cyanobacterial heterocyst glycolipid colocalizes with the cyanobacterial chlorophyll (Fig. 5 and 8B). The heterocyst biosynthetic gene cluster was identified by running antiSMASH on the metaSPAde assembly of the short-read data set. The gene cluster identified by using antiSMASH is shown as the query sequence, and the gene cluster previously deposited in antiSMASH corresponds to the gene cluster named BGC0000869_c1.

**FIG 8:**
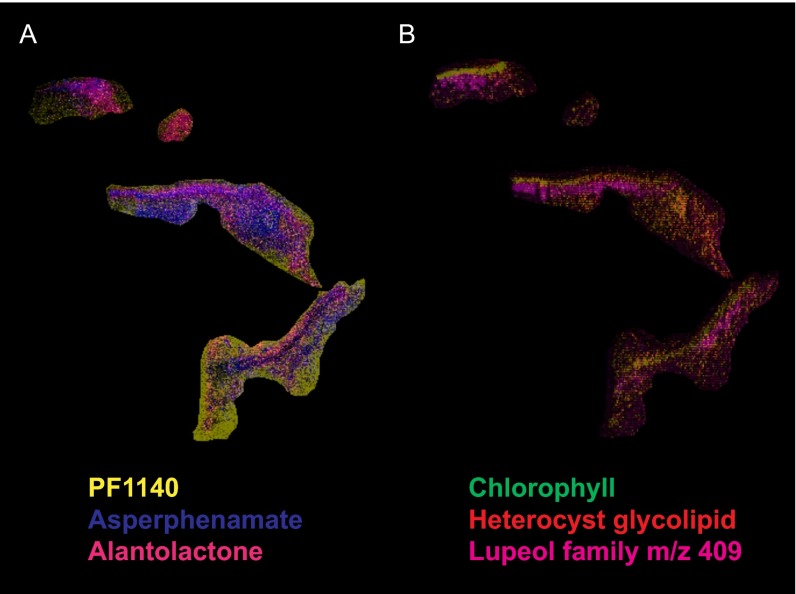
The distribution of fungal molecules PF1140, asperphenamate, and alantolactone** **(A) and cyanobacterial molecules (chlorophyll and heterocyst glycolipid) (B) and a representative member of the molecular family of compounds with spectral similarity to lupeol is shown. The complete overlap of cyanobacterial chlorophyll pigment (green) and heterocyst glycolipid (red) results in cyanobacteria appearing yellow.

Cyanobacteria, being the photobionts in the lichen community, carry out photosynthesis and sugar production, which serve as carbon sources for heterotrophic community members (i.e., fungi and bacteria). Cyanobacterial localization was visualized by the distribution of pheophytin A and pheophorbide A in different layers (Fig. 6; see also Fig. 8B, green). As expected, the highest abundance of these chlorophyll pigments is observed in the sun-exposed top layer. The cyanobacterial layer was also observed using fluorescence microscopy by exciting the sun-exposed layer with green light and detecting the emission of red light (see Fig. S8H in the supplemental material) and overlapped the MS-based distributions of pheophytin A and pheophorbide A shown in Fig. 6. The distribution of a glycolipid, a known cyanobacterial molecule (50) (Fig. 7 and 8B), overlapped the distribution of chlorophyll, further supporting the source of this glycolipid in lichen as being of cyanobacterial origin. Furthermore, the gene cluster for the biosynthesis of the cyanobacterial glycolipids is known and was found in the metagenome of the lichen under study using antiSMASH (51), which provided an additional layer of support for the origin of these molecules (Fig. 7). Thus, combining the metagenomic sequencing-based genotype with the observed chemotype as well as spatial colocalizations of cyanobacterial chlorophyll with heterocyst glycolipid enabled us to assign molecular layers to their respective source. In Fig. 8B, overlapping distribution of chlorophyll and the glycolipid is observed in yellow since chlorophyll was mapped in green and the glycolipid was mapped in red. Distribution of a lichen-associated sterol molecular family putatively annotated as lupeol was visualized at *m/z* 409 (Fig. 8B, pink; see also Fig. S9 in the supplemental material). This compound shows differential distributions with respect to chlorophyll pigments but distribution similar to the alantolactone family of molecules representing different layers of the microbial community. Thus, MS-based molecular networking together with MALDI IMS complements the FISH analysis (22) and enables visualization of multiple microbes within a complex sample based on their molecular signatures.

10.1128/mSystems.00139-16.9 Figure S8  (A to G) MALDI IMS images of adjacent slices that were sliced 10 µm apart. (H) Fluorescence microscopy image of *Peltigera* sp. lichen under study. The distribution of fluorescence matches the distribution of chlorophyll pigments visualized by MALDI IMS in Fig. 4. Download Figure S8, TIF file, 2.1 MB. Copyright © 2016 Garg et al. 2016 Garg et al. This content is distributed under the terms of the Creative Commons Attribution 4.0 International license.

10.1128/mSystems.00139-16.10 Figure S9  The molecular network of the family of a triterpene and a phytosterol, lupeol, is shown. All the labeled peaks in the spectra of lupeol acetate, lupeol, and the insource fragment at *m/z* 409.381 match the fragments of MS/MS spectra available in the METLIN metabolite database. Based upon the fragmentation pattern and spectral match with reference spectra in METLIN, this family is putatively assigned to triterpene-lupeol. Download Figure S9, EPS file, 1.1 MB. Copyright © 2016 Garg et al. 2016 Garg et al. This content is distributed under the terms of the Creative Commons Attribution 4.0 International license.

The multiple molecular layers, indicative of different cellular forms, visualized in the lichen assemblage here are a common occurrence in any multicellular community. For example, the human skin has multiple layers of different cells. The outer layer, namely, the epidermis, provides protection from entry of pathogens and maintains moisture and heat. The middle layer, the dermis, contains sweat and oil glands as well as epithelial cells and blood vessels that provide nourishment and remove waste. Finally, the bottom layer, the hypodermis, contains immune cells such as macrophages that fight infection and invasion by pathogens, adipose tissue, and connective tissue that connects the underlying bone and muscle. This chemical view is consistent with a recent finding published (14) while the manuscript was being prepared that lichens have two fungal layers, an ascomycete and a basidiomycete in the outer cortex layer in addition to the cyanobacterial layer between the cortex and medullary fungal layers. Such an interplay of different cells serving different functions and together functioning as one unit can be imagined for lichen assemblages as well and provides a picture of how different microorganisms come together to form an organized and structured polymicrobial community. The major roles of fungi in the lichen community based on the detected chemistry would be to provide defense and structural support and to store moisture, while the cyanobacteria generate carbon and energy sources. This is exemplified in the lichen by the embedding of the cyanobacterial layer between the cortical and medullary fungal layers, as visualized by optical microscopy (23) and in this study by MALDI IMS-based visualization of chlorophyll pigments. Although direct MS-based visualization of an environmental sample is now possible, understanding the complex chemical interactions involves the arduous task of identifying specialized metabolites among thousands of detected molecules. We identified different classes of molecules belonging to fungi, cyanobacteria, and bacteria through the use of GNPS infrastructure. GNPS enabled the use of publicly available metabolomics data sets representative of different lichen community members, which made it possible to assign taxonomy to molecules produced by the contributors within the complex community. Furthermore, isolation of fungi and cyanobacteria from the lichen sample allowed comparison of molecules produced under culture conditions with those that were detected directly from the lichen sample. Visualization of molecular layers using MALDI IMS suggested that a lichen comprises different cellular layers. Such an approach where one can define the chemotype by metabolomics and the genotype by metagenomics sequencing and can visualize chemotypes by IMS can be applied not only to environmental communities such as lichen, marine sponges, and algae but also to studying the role of the human microbiome in health and disease, representing a broad applicability of this methodology.

### Conclusion.

Lichens represent complex microbial consortia, which grow in highly specialized environments such as in rainforests and in isolated spots of the natural world that are too harsh or limited for most other organisms. The complex organism diversity contained within the spatially limited lichen thallus is highly likely to provide complex molecular communication between both prokaryotic and eukaryotic community members. By collective use of several strategies in parallel, including MALDI IMS, molecular networking, and metagenomic sequencing, we showed a rich chemical diversity representative of all major organisms in this community. The chemical diversity included peptidic molecules such as asperphenamate, alkaloids such as PF1140, chromophores such as chlorophyll, morphology-associated cyanobacterial glycolipids, triterpene family molecules such as lupeol, and sugars as well as polysaccharides. Based on the distribution of fungal and bacterial molecules, different spatial layers of microbial communities were visualized. Further, these distributions were used to infer roles of the community members. Based on the inferred roles of annotated molecules, fungi and heterotrophic bacteria provide protection by secreting defense molecules, whereas cyanobacteria provide an energy source by converting light into carbon sources which can be utilized by the heterotrophic community members—fungi and the bacteria. Such inferences can be used to understand community interactions and allowed us to identify the origin and biological role of chemical cues, enhancing our understanding of a complex biological phenomenon.

## MATERIALS AND METHODS

### Collection and identification of lichens.

*Peltigera hymenina* was collected from a north-facing grassy slope near the campus of the University of British Columbia (49.2581, −123.2289) and air dried naturally. A portion was deposited in the university herbarium (University of British Columbia, Canada). Identification was based on morphology and comparison of ITS and *rbcLX* sequences obtained using standard PCR techniques. This identification was also consistent with publicly available sequences in GenBank.

### Metagenomic sequencing of *P. hymenina* lichen.

Total DNA was extracted from lichen samples as follows. The surface of the lichen sample (1 cm by 1 cm) was carefully washed 3 times in a sterile tube with 1 ml of nuclease-free and sterile water. After the final wash, the lichen sample was transferred to a clean microcentrifuge tube provided in the Power Soil DNA extraction kit (Mo Bio Laboratories, Inc., Carlsbad, CA). DNA was extracted according to the manufacturer’s instructions. DNA concentration was determined by using the Qubit dsDNA dBR assay kit (Thermo Fisher Scientific, San Diego, CA). A paired-end sequencing library was prepared using the Nextera XT DNA library preparation kit (Illumina, San Diego, CA), and sequencing was performed by the University of California San Diego Sequencing core by using an Illumina MiSeq platform (Illumina) (250-bp paired-end reads). Paired-end sequence data were downloaded from the BaseSpace application (Illumina, San Diego, CA). Raw sequence reads were quality trimmed and filtered using CLC workbench software v. 6.0.1 (CLCbio, Aarhus, Denmark). The following CLC parameters were applied during paired-read sequence trimming and quality control: quality score setting, NCBI/Sanger or Illumina pipeline 1.8 and later; minimum distance, -c quality score 20, -f Phred quality score 33, -m minimum length of sequence to keep after filtering 180 bp. The trimmed reads were subjected to sequence assembly by using the CLC workbench (CLCbio). Open reading frame (ORF) calling and annotations were performed on the contigs obtained from the CLC workbench according to the J. Craig Venter Institute in-house metagenomics reports (METAREP) Web 2.0 application (26). Metagenome annotations were uploaded for taxonomic, functional, and comparative analyses with already-deposited METAREP annotations deriving from metagenomes representing a wide range of environments (e.g., coral, human oral cavity, soils, and lakes) (26). Classification of assembled contigs was performed by using the MG-taxa program with default settings (52). MG-taxa does not rely on homology or sequence alignment; rather, it compares sequence compositions between different taxonomic units. Cluster analyses of annotations and environments were performed using the Multiple Experiment Viewer (MeV) (version 4.8.1) (http://mev.tm4.org/#/welcome) with the average linkage setting and Pearson correlation as distance measure. Due to the high number of taxonomically unassigned genes and contigs, classification of filtered sequence reads was also done by using the BLASTN program (E value cutoff, 1E−5) (53). The total number of metagenome sequence reads obtained from paired-end sequencing was 41,361,470. Quality trimming and metagenome assembly by using the CLC workbench resulted in 549,398 contigs. A total of 136,217 contigs were longer than 300 bp and were included in the downstream analyses. A total of 599,857 genes were detected using the JCVI prokaryotic pipeline, and the METAREP program (54) successfully assigned 463,823 of these genes with functions by using the KEGG annotation tool (see Fig. S3 in the supplemental material). METAREP provided taxonomic classification for cellular organisms (archaea, bacteria, and Eukaryota) of 246,603 genes; however, the remaining 352,254 genes were either unassigned or unresolved. Due to the large number of taxonomically unassigned genes (352,254) in METAREP, the MG-taxa method, which also targets assembled viral metagenomic sequences, was applied (52).

In order to identify biosynthetic gene clusters, metaSPAdes (55), version 3.8.2, was run with default parameters (iterative assembly with k-mer sizes 21, 33, and 55). The metagenomic data set had 20.5 million paired-end reads with a read length of 250 bp and an insert size of 230 bp (13× coverage), and metaSPAdes assembled it in 12,865 contigs larger than 1,000 bp, with *N*_50_ of 22.8 kb, total assembly size of 136 Mb, and largest contig size of 553 kb. AntiSMASH pipeline version 3.0.4 was run on contigs longer than 15 kb with all other options set to default values (https://antismash.secondarymetabolites.org/). AntiSMASH discovered 19 biosynthetic gene clusters in the metagenome, including 8 polyketides, 5 terpenes, three nonribosomal peptides (NRPs), two ribosomally synthesized and posttranslationally modified peptides (RiPPs), and an NRP synthase polyketide synthase (NRPS-PKS). Three of the biosynthetic gene clusters were similar to known natural product gene clusters, one with 100% similarity to geosmin, one with 92% similarity to nosperin, and one with 85% similarity to heterocyst glycolipid.

### Preparation of lichen specimen for MALDI IMS.

The rhizines were removed from the lichen with sterile forceps, and the lichen was completely dried. A 3-cm by 1.8-cm section was cut off from the whole piece of lichen with sterile scissors. A 2.5-mm by 1.8-mm by 3-mm lichen block was excised from this section with a sterile scalpel for analysis by MALDI IMS. An additional block 10 µm away was also sliced to confirm observed molecular distributions from the adjacent sections. The rest of the lichen sample was placed in a petri dish and fixed with metal clips. A BD PrecisionGlide 1.6-mm by 40-mm needle was used to scrape off 110 small spots from the lichen surface.

### Microbial cultivation from *P. hymenina* lichen.

A small piece of lichen specimen was transferred to a sterile Falcon tube, 10 ml of sterile Milli-Q water and glass beads were added, and the sample was vortexed for 1 min. Different solid agar media were used for microbial isolation: sterile nutrient solution (SNS) (56), Luria broth (LB), NZ amine broth (NZY), and *Cyanobacteria* BG-11 freshwater solution (Sigma) (CBG). LB, SNS, and CBG were prepared with sterile Milli-Q water and supplemented with 50 mg/liter of both cycloheximide and nalidixic acid. Another set of LB isolation plates was prepared with 50 mg/liter of either cycloheximide or nalidixic acid. The vortexed lichen specimen was plated using two different methods: (i) the mixture was serially stamped onto solid agar with a sterile swab or (ii) the mixture was diluted with 1 ml of sterile Milli-Q water and 100 µl of the resulting mixture was spread onto the plate surface. Cultures were incubated at room temperature, and microbial colonies were subcultured on either ISP2 or CBG in the case of suspected cyanobacterial photosymbionts. Typical incubation times for the appearance of colonies from isolation plates ranged from 10 to 90 days.

### MALDI IMS.

The excised samples for MALDI IMS were embedded in 25 mg/ml of gelatin solution and placed on an isopropanol-dry ice bath to prevent diffusion of metabolites during freezing. The frozen blocks were kept in the cryostat (Leica BM CM1850) at 21°C for 2 h. After 2 h, each of the two blocks was sectioned into three 12-µm-thick sections and each section was transferred to an indium tin oxide (ITO) conductive glass slide (Bruker Daltonik GmbH). The sections on the ITO-coated glass slide were dried overnight and covered with 2,5-dihydroxybenzoic acid MALDI matrix (Sigma-Aldrich) applied by sublimation. The apparatus and the procedure used for matrix deposition by sublimation were described previously (57). Briefly, the matrix (300 mg) was added to the base section of the sublimator and dissolved in acetone. The dissolved matrix was dried under nitrogen while the sublimator was continuously swirled to form a thin layer of matrix at the base of the sublimator. The apparatus was assembled, and the condenser was connected to an ice bath. The system was placed under vacuum for 20 min. After 20 min, the ITO glass slide with the sample was affixed to the base of the condenser, and vacuum was applied for another 10 min. The heating mantle at 40°C was now placed under the base of the sublimator for 7.5 min. The matrix-covered ITO glass slide was carefully removed and imaged in the reflectron positive mode from *m/z* 200 to 2,000 using a Bruker Autoflex Speed MALDI mass spectrometer (Bruker Daltonik GmbH, Bremen, Germany) with 15-µm raster steps and 200 shots per raster location using the random walk shot pattern at a laser power of 60%. The data analysis was performed using flexControl (version 3.0; Bruker Daltonik GmbH) and flexImaging (version 3.0; Bruker Daltonik GmbH) software. The instrument was calibrated prior to data acquisition using a peptide calibration standard (Bruker Daltonik GmbH). The data acquired on an adjacent block are shown in Fig. S8A to H in the supplemental material.

### Collection of tandem MS data and molecular networking.

Metabolites from each of the individual 110 spots were extracted with 4:1 ethyl acetate-methanol and 0.1% trifluoroacetic acid (TFA). The samples were sonicated for 10 min in a water bath and held at room temperature for 30 min. The microbial cultures were extracted using the same extraction conditions. A small plug of agar was removed with a sterile scalpel and placed in an Eppendorf tube containing 200 µl of 4:1 ethyl acetate-methanol and 0.1% TFA. The extracted metabolites from lichen and microbial cultures were dried under vacuum in a lyophilizer. The dried extracts were resuspended in 100% acetonitrile. The resuspended extracts were analyzed with an UltiMate 3000 UHPLC system (Thermo Scientific) using a Kinetex 1.7-µm C_18_ reversed-phase UHPLC column (50 by 2.1 mm) and a Maxis Q-TOF mass spectrometer (Bruker Daltonics) equipped with an electrospray ionization (ESI) source. For chromatographic separation, the column was kept at 2% solvent B (98% acetonitrile, 0.1% formic acid in LC-MS-grade water) and 98% solvent A (0.1% formic acid in water) for 1 min, followed by a linear gradient reaching 99% solvent B in 16 min. The column was then held at 99% B for 1.5 min, brought back to 2% solvent B in 0.5 min, and kept under these conditions for 2 min. The column was then washed by bringing it back to 99% in 1 min, followed by equilibration to an initial condition of 2% solvent B over 1 min. The chromatography was performed at a flow rate of 0.5 ml/min throughout the run. MS spectra were acquired in positive ion mode in the mass range of *m/z* 100 to 2,000. An external calibration with ESI-L low-concentration tuning mix (Agilent Technologies) was performed prior to data collection and internal calibrant hexakis(1*H*,1*H*,3*H*-tetrafluoropropoxy)phosphazene was used throughout the runs. The capillary voltage of 4,500 V, nebulizer gas pressure (nitrogen) of 160 kPa, ion source temperature of 200°C, dry gas flow of 7 liters/min at source temperature, and spectral rate of 3 Hz for MS^1^ and 10 Hz for MS^2^ were used. For acquiring MS/MS fragmentation, the 10 most intense ions per MS^1^ were selected. Basic stepping function was used to fragment ions at 50% and 125% of the collision-induced dissociation (CID) calculated for each *m/z* (33) with a timing of 50% for each step. Similarly, basic stepping of collision radio frequency (RF) of 550 and 800 volts peak to peak (Vpp) with a timing of 50% for each step and transfer time stepping of 57 and 90 µs with a timing of 50% for each step was employed. The MS/MS active exclusion parameter was set to 3 and was released after 30 s. The mass of internal calibrant was excluded from the MS^2^ list. Molecular network analysis was performed at GNPS, and the analysis parameters, networking statistics, and network summarizing graphs are available at http://gnps.ucsd.edu/ProteoSAFe/status.jsp?task=675991870933480293c10f0bfcf69e20. This data set is accessible from the MassIVE repository, and the associated accession number is MSV000078584. The stereochemistry of the structures drawn in the figures is from published literature and was not determined in this work.

### Accession number(s).

Lichen metagenomic reads have been submitted to NCBI under BioProject ID PRJNA317389. The *P. hymenina* ITS NCBI accession number is KX790924. The lichen LC-MS accession number is MSV000078584. The isolate LC-MS accession number is MSV000080115.
