# Supplementary material for: Spatial Molecular Architecture of the Microbial Community of a Peltigera Lichen
Source: mSystems. 2016 Dec 20;1(6):e00139-16. doi: 10.1128/mSystems.00139-16 (PMC5183598; doi:10.1128/mSystems.00139-16)
Supplement: Text S1 [file sys006162074s1.docx]

**The Spatial Molecular Architecture of the Microbial Community of Peltigera Lichen.**

Neha Garg^1*^, Yi Zeng^2*^, Anna Edlund^3^, Alexey V. Melnik^1^, Laura Sanchez^4,^, Hosein Mohimani^11^**,** Alexey Gurevich^12^, Vivian Miao^5^, Stefan Schiffler^6^, Yan Wei Lim^7^, Tal Luzzatto-Knaan^1^, Shengxin Cai^8^, Forest Rohwer^7^, Pavel A. Pezner^11^, Robert H. Cichewicz^8^, Theodore Alexandrov^1,6,9^, and Pieter C. Dorrestein^1,2,10,#^

**Running Title: Molecular organization in a lichen**

**Text S1.** Supplementary text.

**Metagenomics Analysis.**

The highest scoring contigs (rank score 1; 500 bp-10,364 bp long) from the MG-Taxa analysis belonged to eukaryotes such as *Apicomplexa*, *Kinetoplastida*, *Oligohymenophorea* (supplementary table S1 available at <ftp://massive.ucsd.edu/MSV000078584/updates/2016-11-18_zengyi88516_edceffc7/other/>). Several interesting viral signatures were observed and an *Amsacta moorei entomopox virus ‘L’*, previously known to infect invertebrates (insects) such as grasshoppers was identified (rank score 0.89*,* ([1](#_ENREF_1))). Six contigs were identified with high ranks scores (0.79 - 0.97) belonging to the methanogen *Methanobrevibacter ruminantium* M1 ([2](#_ENREF_2)) showing that the *Peltigera* hymenina can serve as a methane source. Also, two contigs (rank scores 0.85; lengths 43,482 and 35,834 bp, respectively) belonging to *Lactococcus phage bIL312* were identified. This prophage was previously identified in deep water samples from the Mediterranean Sea ([3](#_ENREF_3)) and classified as virulent based on findings from a comparative genomics study of prophages in the *Lactococcus lactis* genome ([4](#_ENREF_4)). In addition, six contigs belonging to Macacine herpesvirus 1 showed high rank scores (0.96) at the species level.

Based on hierarchical cluster analyses of the taxonomic annotations obtained from METAREP, the lichen was most similar to soil metagenomes and orders of bacteria specifically belonging to the *Actinomycetales*, *Rhizobiales*, *Sphingomonadales*, *Burkholderiales*, *Nostocales*, *Acidobacteriales* were shared between the two environments (supplementary Figure S2).

**Annotation of molecules.**

Using the GNPS reference library, a number of MS/MS spectra matched to known molecules. These are level two annotation according to the 2007 metabolomics standards initiative ([5](#_ENREF_5)).

The pyridone alkaloid PF1140 was identified in the MS/MS spectra acquired on *Peltigera* sp. lichen (Figure 2A) and the isolated microbes by spectral matching to the MS/MS spectra available in the GNPS library that was acquired in-house. Using the molecular network we also identified an analogue of PF1140 in the extract of cultured microbe (reported in literature as deoxy-PF1140) (Figure 2B) where the connecting nodes differed by a delta mass of 15.99 Da. The fragments at *m/z* 124.034, 166.085, and 192.100 in deoxy-PF1140 also differed in mass by 15.99 Da from the fragments at *m/z* 140.035, 182.081, and 208.097 in PF1140 further supporting that this analogue is deoxy-PF1140. Interestingly, the molecular network also contained a node at *m/z* 354.207, which is 92.03 Da higher in mass than deoxy-PF1140 consistent with the suggesting addition of a phenol moiety. The fragments at *m/z* 216.065, 258.112, and 284.112 are shifted by 92.03 Da in mass from the corresponding fragments of deoxy-PF1140 (dashed lines in Figure 2C). Indeed a known molecule, namely trichodin A ([6](#_ENREF_6)), has phenol group attached to pyridone ring of demethylated deoxy-PF1140. Based on structure of PF1140, the methyl group is putatively shown on the position 8. The node at *m/z* 280.193 likely represents the known pyridone alkaloid deoxy-akanthomycin based on the common fragments at *m/z* 262.181, 192.101, 166.085, and 124.040 in MS/MS spectrum ([7](#_ENREF_7)). The unknown pyridone alkaloid at *m/z* 372.216 with 92.03 Da mass increase from putative deoxyakanthomycin would involve addition of phenol group to deoxyakanthomyin (supplementary Figure S5). Similar to the asperphenamate analogues, only one of the several pyridone alkaloids produced in culture was directly detected from the lichen. Although it is common for one gene cluster to make many molecules ([8](#_ENREF_8)), this suggests that microbes in the community control the types of molecules that are produced further suggesting that microbes regulate their chemical response in community structure.

The fungal molecule, asperphenamate ([9](#_ENREF_9)), which was identified in the fungal public dataset MSV000079098, was also found in the MS/MS dataset of lichen and one of the cultured isolates (Figure 3). Out of the 462 of the extracts analyzed in the public dataset MSV000079098, asperphenamate was identified in 33 of extracts. One of these 33 extracts belonged to an *Alternaria* sp. fungus by 18S rRNA sequencing. The match to asperphenamate was confirmed by annotating the MS/MS fragments at *m/z* 238.123, 256.133, 224.107, and 105.033 Da, also present in the reference spectra of asperphenamate in the GNPS library that was acquired in-house. Two previously reported analogues of asperphenamate at *m/z* 523.222 that has phenylalanine to tyrosine modification (confirmed by fragment at *m/z* 268.097) and *m/z* 508.223 with replacement of CH with N atom to the left of the ester bond was identified in the microbial isolate but not in the lichen. Molecular networking also showed production of a previously unknown new analogue of asperphenamate at *m/z* 564.250. The delta *m/z* of 57.02 Da between nodes is consistent with a molecular formula the size of glycine. Annotation of fragments at *m/z* 309.124, and the fragments *m/z* 188.072, and 160.075 in the MS/MS spectra confirmed this additional mass the left of the ester bond (Figure 3).

The unannotated molecular masses derived from the network analysis were also searched against public databases. A molecule at *m/z* 233.154 was identified as a match to sesquiterpene lactone namely, alantolactone by searching METLIN metabolite database. The transition of *m/z* 233.154 to *m/z* 105.069 for alantolactone ([10](#_ENREF_10)) and 249.148 to 231.136 for hydroxyalantolactone ([11](#_ENREF_11)) has been previously published further supporting annotation of this molecular family (Figure 4). Various other analogues of alantolactone annotated using METLIN database are also represented in Figure 4. Identification of such molecules in lichen further highlights complex chemical interactions that take place between its community members and the environment as well as ancient realization of the significance of lichens in medicinal drug discovery.

The sugar mannitol (Figure 5) and UDP-N-acetylglucosamine (Figure S7) was identified by molecular networking in our lichen sample. Spectral match to the library spectra in GNPS was confirmed by acquiring tandem mass spectra on commercial standards of both sugars. The molecular families corresponding to polysaccharides were searched using the network summarizing graphs analysis in GNPS for common mass shifts of 144, 162, 176, and 324 Da corresponding to sugar residues (supplementary Figure S6A). The molecular network shown in supplementary Figure S6B fit this category with delta masses between nodes suggestive of sugar residues. The MS/MS spectra for some of these molecules had a fragment at *m/z* 183.09, which is the predicted molecular mass of mannitol. Furthermore, these spectra had all the fragments that were detected in the spectra of mannitol itself (Figure 5). In addition, neutral loss of 162.06 Da in the MS/MS spectra of polysaccharide with *m/z* 345.138 is consistent with an additional hexose residue (Figure 5). Similarly, the polysaccharide at *m/z* 689.272 contains two additional hexose residues.

The second polysaccharide family consisted of polyacetylated hexose residue (neutral loss of 162.06 + n×42.01 Da) and a mass loss that is consistent with glucuronic acid (loss of 176.07Da) (supplementary Figure S7). The connecting nodes in this molecular family differed by 42.01 suggesting multiple acetylations on the sugar residues (supplementary Figure S6C and S7). Multiple O-acetylations of sugar units have been reported for *O*-specific polysaccharide of a fish pathogen, *Aeromonas bestiarum* ([12](#_ENREF_12)).

The cyanobacterial photosynthetic pigments pheophytin A and pheophorbide A were dereplicated by spectral match to the library spectra in GNPS (Figure 6). The dominant fragments at *m/z* 533.254 and 593.278 are annotated and were observed in the spectra of both the commercial standard and the lichen. The cyanobacterial glycolipids were identified at *m/z* 577.467 and *m/z* 575.452. The MS/MS spectra matched completely the published spectra of cyanobacterial glycolipids 1-(*O*-hexose)-3,25-hexacosanediol and 1-(*O*-hexose)-3-keto-25-hexacosanol (Figure 7) isolated from *Anabaena* CCY9614 ([13](#_ENREF_13)). These glycolipids are associated with growth of cyanobacteria as heterocysts, a form that is observed in lichens ([14](#_ENREF_14)).

A molecular family corresponding to a sterol fragmentation pattern was also observed. This sterol pattern most closely resembled the triterpene lupeol (isolated from medicinal plants and vegetables ([15](#_ENREF_15)) with a series of low abundance and low *m/z* fragment ions at with mass difference of 14.02 Da between fragments was also identified by manually searching the METLIN metabolite database (supplementary Figure S9). The MS/MS spectra of molecules at *m/z* 469.4 (putative lupeol acetate), *m/z* 427.391 (consistent with lupeol), and in-source fragment at *m/z* 409.384 matched with the MS/MS spectra available in METLIN.

**References.**

1. **Bawden AL, Glassberg KJ, Diggans J, Shaw R, Farmerie W, Moyer RW.** 2000. Complete genomic sequence of the Amsacta moorei entomopoxvirus: analysis and comparison with other poxviruses. Virology **274:**120-139.

2. **Leahy SC, Kelly WJ, Altermann E, Ronimus RS, Yeoman CJ, Pacheco DM, Li D, Kong Z, McTavish S, Sang C, Lambie SC, Janssen PH, Dey D, Attwood GT.** 2010. The genome sequence of the rumen methanogen Methanobrevibacter ruminantium reveals new possibilities for controlling ruminant methane emissions. PLOS ONE **5:**e8926.

3. **Winter C, Garcia JA, Weinbauer MG, DuBow MS, Herndl GJ.** 2014. Comparison of deep-water viromes from the atlantic ocean and the mediterranean sea. PloS one **9:**e100600.

4. **Chopin A, Bolotin A, Sorokin A, Ehrlich SD, Chopin M.** 2001. Analysis of six prophages in Lactococcus lactis IL1403: different genetic structure of temperate and virulent phage populations. Nucleic acids research **29:**644-651.

5. **Sumner LW, Amberg A, Barrett D, Beale MH, Beger R, Daykin CA, Fan TW, Fiehn O, Goodacre R, Griffin JL, Hankemeier T, Hardy N, Harnly J, Higashi R, Kopka J, Lane AN, Lindon JC, Marriott P, Nicholls AW, Reily MD, Thaden JJ, Viant MR.** 2007. Proposed minimum reporting standards for chemical analysis Chemical Analysis Working Group (CAWG) Metabolomics Standards Initiative (MSI). Metabolomics : Official journal of the Metabolomic Society **3:**211-221.

6. **Wu B, Oesker V, Wiese J, Schmaljohann R, Imhoff JF.** 2014. Two new antibiotic pyridones produced by a marine fungus, Trichoderma sp. strain MF106. Marine drugs **12:**1208-1219.

7. **Wagenaar MM, Gibson DM, Clardy J.** 2002. Akanthomycin, a new antibiotic pyridone from the entomopathogenic fungus Akanthomyces gracilis. Organic letters **4:**671-673.

8. **Fischbach MA, Clardy J.** 2007. One pathway, many products. Nature chemical biology **3:**353-355.

9. **Kildgaard S, Mansson M, Dosen I, Klitgaard A, Frisvad JC, Larsen TO, Nielsen KF.** 2014. Accurate dereplication of bioactive secondary metabolites from marine-derived fungi by UHPLC-DAD-QTOFMS and a MS/HRMS library. Marine drugs **12:**3681-3705.

10. **Guo C, Zhang S, Teng S, Niu K.** 2014. Simultaneous determination of sesquiterpene lactones isoalantolactone and alantolactone isomers in rat plasma by liquid chromatography with tandem mass spectrometry: application to a pharmacokinetic study. Journal of separation science **37:**950-956.

11. **Yang X, Su J, He Y, Liu H, Li H, Zhang W.** 2012. Simultaneous determination of three sesquiterpene lactones from Herba Inula extract in rat plasma by LC/MS/MS and its application to pharmacokinetic study. J Chromatogr B Analyt Technol Biomed Life Sci **903:**40-45.

12. **Turska-Szewczuk A, Lindner B, Komaniecka I, Kozinska A, Pekala A, Choma A, Holst O.** 2013. Structural and immunochemical studies of the lipopolysaccharide from the fish pathogen, Aeromonas bestiarum strain K296, serotype O18. Marine drugs **11:**1235-1255.

13. **Bauersachs T, Hopmans EC, Compaore J, Stal LJ, Schouten S, Damste JS.** 2009. Rapid analysis of long-chain glycolipids in heterocystous cyanobacteria using high-performance liquid chromatography coupled to electrospray ionization tandem mass spectrometry. Rapid communications in mass spectrometry : RCM **23:**1387-1394.

14. **Henskens FL, Green TG, Wilkins A.** 2012. Cyanolichens can have both cyanobacteria and green algae in a common layer as major contributors to photosynthesis. Annals of botany **110:**555-563.

15. **Siddique HR, Saleem M.** 2011. Beneficial health effects of lupeol triterpene: a review of preclinical studies. Life sciences **88:**285-293.
