# Supplementary figures and images for: Spatial Molecular Architecture of the Microbial Community of a Peltigera Lichen
Source: mSystems. 2016 Dec 20;1(6):e00139-16. doi: 10.1128/mSystems.00139-16 (PMC5183598; doi:10.1128/mSystems.00139-16)

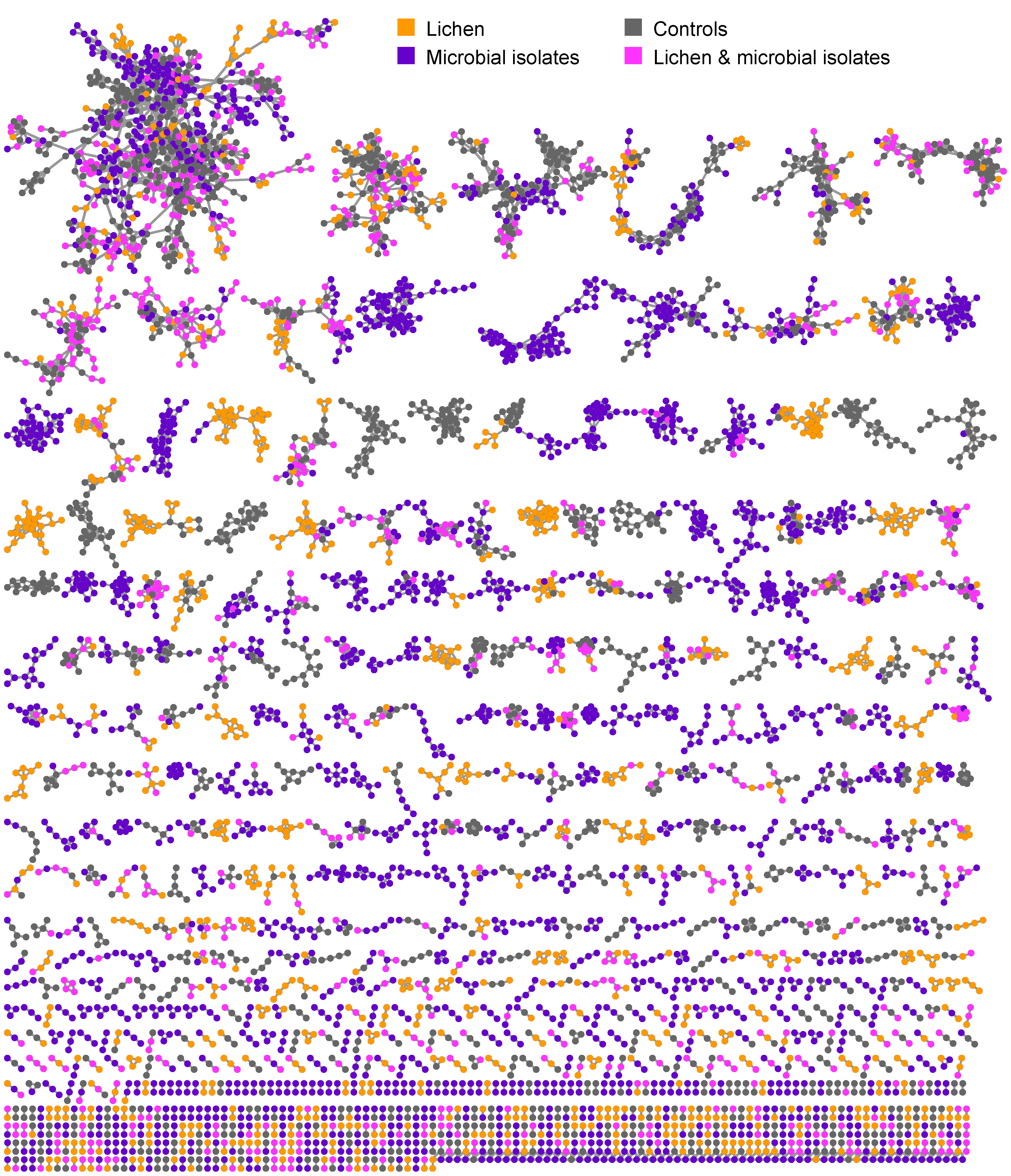

Supplement: Figure S4 [file sys006162074sf5.tif]

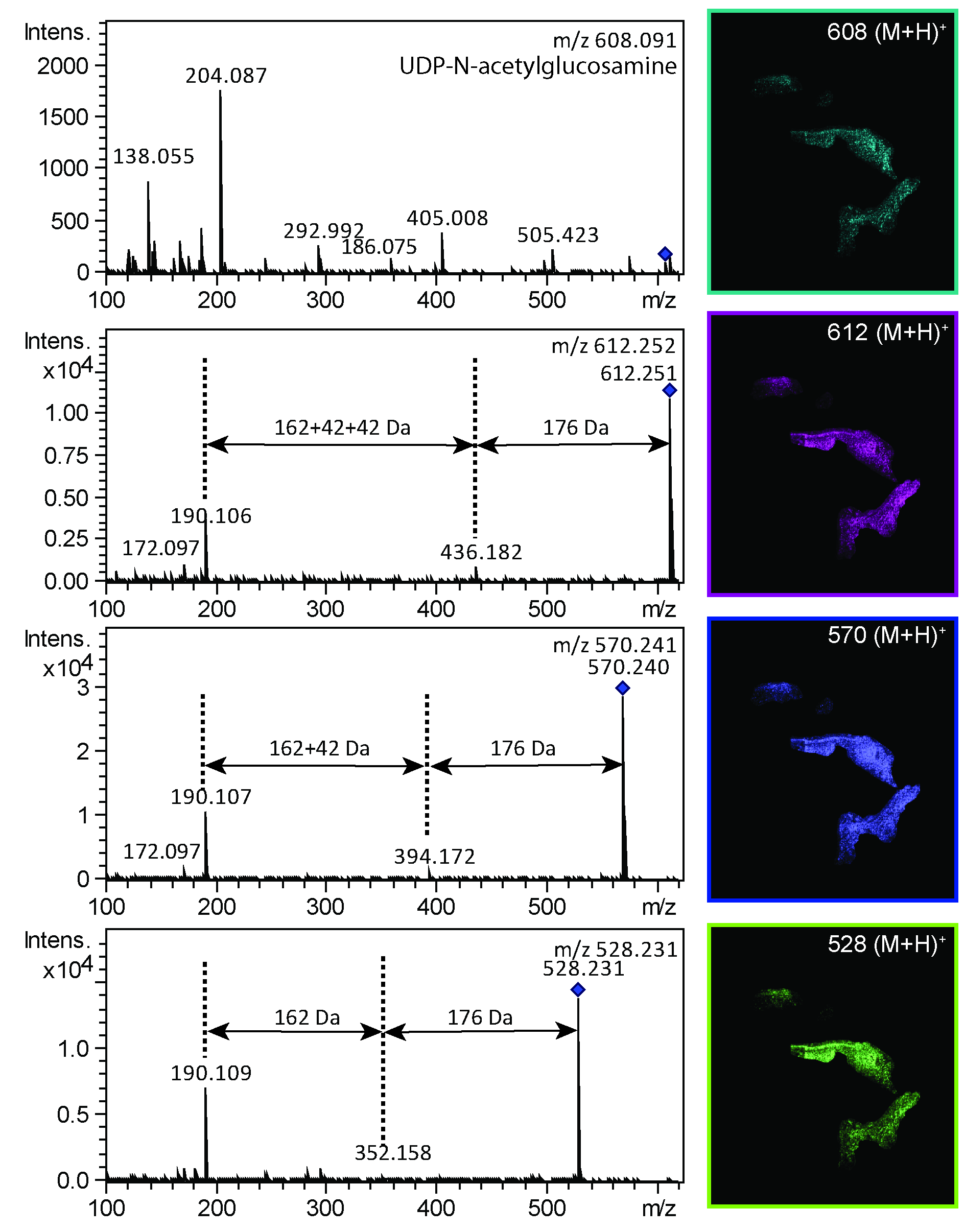

Supplement: Figure S7 [file sys006162074sf8.tif]

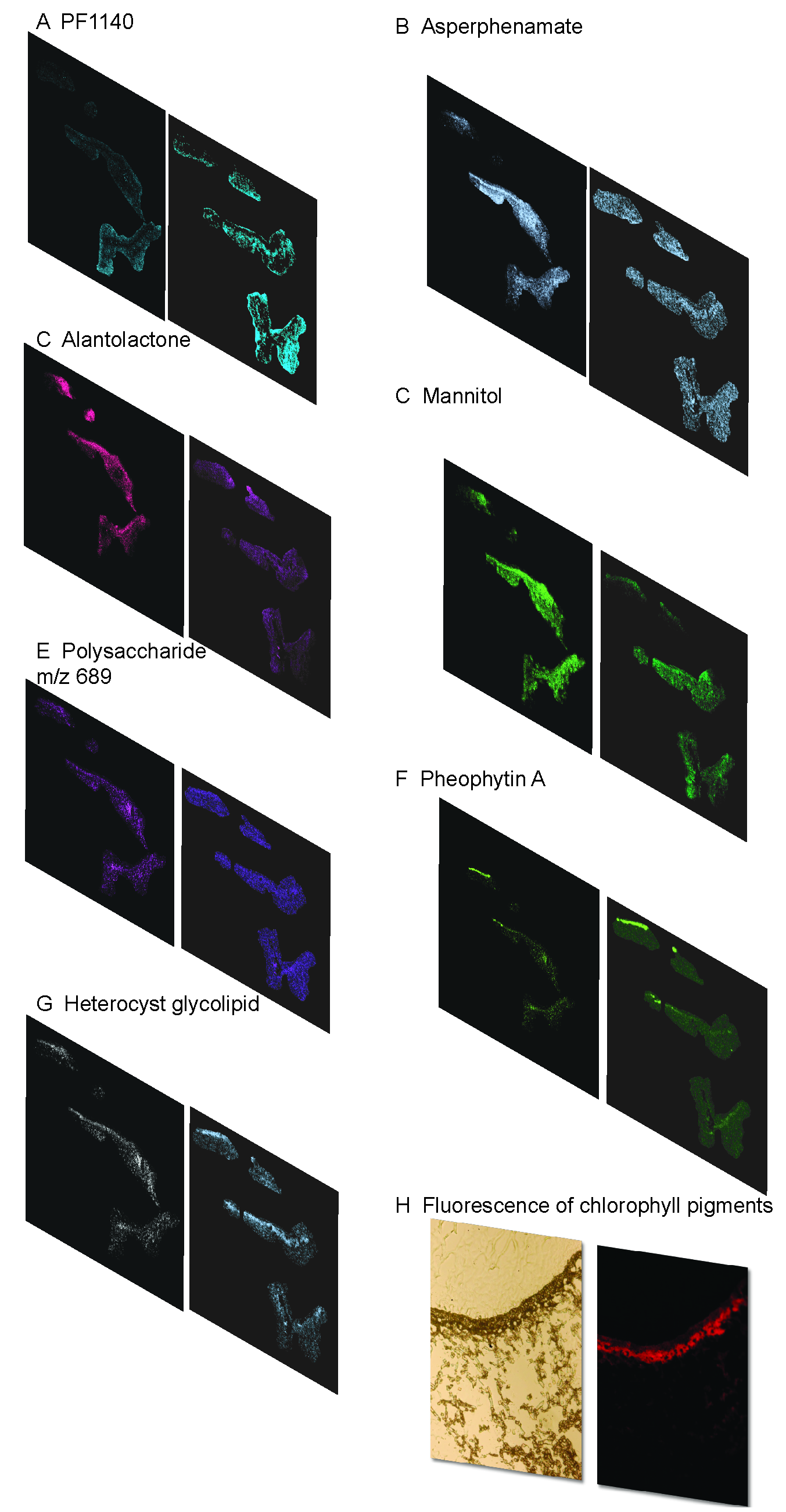

Supplement: Figure S8 [file sys006162074sf9.tif]
